# CuATSM Enhances Wound Repair Without Scarring via Hippo/YAP Signalling Pathway to Reduce Ferroptosis and Macrophage Polarisation

**DOI:** 10.1111/jcmm.70590

**Published:** 2025-06-17

**Authors:** Yingdan Tang, Jiazong Ye, Tingye Xu, Yuhuan Sun, Weiyang Meng, Lielie Zhu, Zhongheng Jia, Qian Wu, Daqing Chen, Fangfang Wu

**Affiliations:** ^1^ Emergency Department, The Second Affiliated Hospital and Yuying Children's Hospital Wenzhou Medical University Wenzhou China; ^2^ Zhejiang Engineering Research Center for Innovation and Application of Intelligent Radiotherapy Technology, Zhejiang‐Hong Kong Precision Theragnostic of Thoracic Tumors Joint Laboratory, Wenzhou Key Laboratory of Basic Science and Translational Research of Radiation Oncology The Second Affiliated Hospital of Wenzhou Medical University Wenzhou Zhejiang China; ^3^ Department of Ultrasound Wenzhou Dongtou District People's Hospital Wenzhou China; ^4^ Wenzhou Medical University Wenzhou China; ^5^ Wenzhou Medical University RenJi College Wenzhou China; ^6^ Department of Rehabilitation Wenzhou TCM Hospital of Zhe Jiang Chinese Medical University Wenzhou China

**Keywords:** CuATSM, ferroptosis, hippo/YAP pathway, scarless, wound healing

## Abstract

Skin wound healing is a complex biological process involving haemostasis, inflammation, proliferation/repair and remodelling. However, skin scarring, as one of the important stages in the healing process, can adversely affect the structure and function of related organs. Currently, effective treatments to address such scars remain insufficient. In this study, we established a full‐thickness skin excision wound model using male ICR mice, which were randomly divided into a Control group and a CuATSM group. The CuATSM group received CuATSM (30 mg/kg) via gavage, with daily treatments continuing throughout the observation period. The Control group received an equivalent volume of 0.9% sodium chloride solution. Wound healing progression was evaluated through macroscopic photography, histological analyses, Western blotting and quantification of relevant biochemical markers at different healing stages. Our study reveals that CuATSM not only promotes rapid skin wound healing but also reduces scar formation in the late healing phase. Furthermore, our findings suggest that this effect is mediated through the ferroptosis‐induced Hippo/YAP signalling pathway and macrophage polarisation. These findings highlight CuATSM as a promising therapeutic candidate for achieving scarless wound repair in clinical applications.

## 1 Introduction

Skin wound healing is a complex and dynamic physiological process divided into four interrelated and overlapping phases: haemostasis, inflammation, proliferation and remodelling. Dysregulation of these phases may impair healing and result in scar formation. Despite significant advances in research over recent decades, most scar prevention and treatment strategies remain clinically suboptimal [1, 2]. Therefore, there is a critical need for drugs that promote wound healing and alleviate scar formation.

A large number of studies have shown that encouraging the transition of macrophages from pro‐inflammatory (M1) to anti‐inflammatory (M2) phenotypes is a crucial strategy for enhancing scarless healing [3, 4]. Meanwhile, excessive fibroblast activity leads to scar formation [5]. Besides, studies have indicated that hair follicles and hair follicle stem cells can reduce scar formation after wound healing [6, 7]. Hence, promoting macrophage polarisation, reducing excessive fibroblast proliferation and increasing the generation of hair follicles and hair follicle stem cells provide new research directions for the treatment of scar formation or pathological scarring.

The Hippo/YAP pathway is an evolutionarily conserved signalling cascade essential for organ development, epithelial homeostasis, wound healing, tissue regeneration and immune modulation [8, 9]. It also plays a pivotal role in fibrotic diseases affecting various organs, such as the lungs, liver, kidneys and heart [10, 11, 12]. The pathway's transcriptional effectors YAP/TAZ regulate gene expression through interaction with TEAD transcription factors [12]. Elevated YAP/TAZ levels have been observed in skin biopsies from patients with systemic sclerosis (SSc), and dimethyl fumarate (DMF), a potent YAP/TAZ inhibitor, has shown efficacy in reducing skin fibrosis [13]. Meanwhile, verteporfin, a YAP protein inhibitor, has been found to significantly reduce cartilage regeneration while enhancing ectopic hair follicle formation in the wound healing models [14]. Therefore, targeting the Hippo/YAP pathway by modulating YAP/TAZ nuclear translocation may be an effective therapeutic approach to promote scarless wound healing.

Ferroptosis represents an iron‐dependent, non‐apoptotic form of programmed cell death [15]. This unique mode of cell death stands apart from classical apoptosis, necrosis and autophagy, and is characterised by iron accumulation, lipid peroxidation and a depletion in the levels of the antioxidant glutathione [16]. Ferroptosis plays an important role in many diseases, such as wound healing, sepsis, the treatment of drug‐resistant cancers, ischemic organ injuries and other degenerative diseases linked to overwhelming lipid peroxidation [17]. Accumulating evidence suggests that ferroptosis is intricately involved in the initiation, progression, therapeutic management and prognostic outcomes of various wound types [18, 19, 20]. Furthermore, inhibiting ferroptosis may help alleviate inflammation, reduce the formation of corneal scars, which can improve the prognosis of Bacterial keratitis (BK). Chen Q et al. found that inhibiting ferroptosis effectively reduces the inflammatory response and corneal scar formation in mouse models of bacterial keratitis [21]. Thus, modulating ferroptosis presents a compelling strategy to promote scarless wound healing.

Copper (II)‐2‐acetylpyridine bis (4‐methyl‐3‐thiosemicarbazone) (CuATSM), a recognised ferroptosis inhibitor, is widely used in clinical applications and neurodegenerative disease research [22, 23]. CuATSM facilitates the delivery of copper ions into cells [24]. Zilka O and colleagues have demonstrated that CuATSM holds promise as a clinical candidate for the treatment of amyotrophic lateral sclerosis and Parkinson's disease, functioning as a highly effective radical‐trapping antioxidant (RTA) and inhibitor of (phosphorylated) lipid peroxidation, while simultaneously safeguarding mouse embryonic fibroblasts and hippocampal cells from ferroptosis [23]. Numerous studies have highlighted the efficacy of Cu^2+^ in promoting wound healing [25, 26, 27], but the potential of CuATSM in regulating ferroptosis and the Hippo/YAP pathway during wound repair remains unexplored. Our study aims to investigate the role of CuATSM in skin wound healing as well as the underlying mechanisms of its action.

In this study, we observed that CuATSM facilitates wound healing through mechanisms including the promotion of macrophage polarisation, enhancement of collagen deposition and induction of hair follicle regeneration. Additionally, it also plays a pivotal role in promoting scarless healing through the regulation of the Hippo/YAP pathway and the suppression of ferroptosis. These actions result in the downregulation of En‐1 and CD26, along with the upregulation of CK19 and Sox9, all of which are critical markers involved in the regenerative processes.

## 2 Materials and Methods

### 2.1 Animal Model and Treatment

ICR mice (5–6 weeks old, male) were used to evaluate the in vivo wound healing effect of CuATSM. All the mice were randomly assigned to either the Control group or the treatment group, with each mouse in the treatment group receiving CuATSM (30 mg/kg) via gavage 2 h before modelling and daily throughout the observation period after modelling. The mice were anaesthetised with 2.5% isoflurane. Then, dorsal hair was removed using an electric shaver and hair removal cream, followed by sterilisation with povidone‐iodine. A 0.5 mm thick rubber gasket was secured onto the backs of the mice using 5–0 surgical sutures, and a full‐thickness circular wound was created within the gasket. Finally, wounds of all mouse groups were covered with a sheet of 3 M Tegaderm Film and were wrapped with a self‐adhesive bandage. Skin wounds were photographed at days 0, 3, 6, 9, 12, 15 and 35 after injury and analysed using ImageJ. The wound area was defined as the region lacking fresh skin tissue growth. The area was outlined by following the clear edge of the wound. The scale was calibrated using a ruler placed adjacent to the wound in each photograph.

### 2.2 Histological Analysis

The mice were sacrificed on the indicated days (14, 28 or 35) after surgery. The skin tissues were fixed in 4% paraformaldehyde for 24 h, embedded in paraffin and 5 μm sections were cut from the paraffin‐embedded blocks. The sections were dewaxed, hydrated and stained with Haematoxylin and Eosin (H&E) and Masson's trichrome staining. H&E staining kits and Masson's trichrome staining kits were obtained from Solarbio (G1121 and G1346, respectively), and used according to the manufacturer's protocols. Finally, the images were captured using a microscope (Nikon, Japan).

### 2.3 Immunofluorescence Staining

The skin tissues from the corresponding days post‐modelling were collected for immunofluorescence staining. The tissues were fixed with 4% paraformaldehyde (PFA) in phosphate‐buffered saline (PBS) for 24 h, and then embedded in paraffin. 5 μm slices were deparaffinised, rehydrated and washed. Antigen retrieval was performed using a citric acid buffer (pH 6.0) in a microwave oven for 2 min. The slices were then permeabilised with 0.1% Triton X‐100 for 15 min. Subsequently, the slices were blocked with 5% bovine serum albumin (BSA) in PBS for 30 min at 37°C before overnight incubation at 4°C with primary antibodies against CD31 (1:500, ab9498, Abcam), GPX4 (1:100, ab125066, Abcam), YAP (1:100, sc‐101199, Santa), EN‐1 (1:100, sc‐398534, Santa), CD26 (1:200, 67138, Cell Signalling Technology), Col I (1:100, 14695‐1‐AP, Proteintech), Sox9 (1:200, ab185966, Abcam), CD206 (1:100, 18704‐1‐AP, Proteintech), CK19 (1:100, ab52625, Abcam). After three washes with PBST, the sections were incubated with Alexa Fluor 488‐conjugated anti‐rabbit secondary antibody (ab150073, Abcam) at a dilution of 1:1000 for 1 h at 37°C. Cellular nuclei were afterwards counterstained with DAPI. Lastly, images were captured using a Nikon ECLIPSE Ti microscope (Nikon, Japan).

### 2.4 Western Blot

The mice were sacrificed on the corresponding days after the operation, and wound tissue was snap‐frozen and stored in liquid nitrogen for use in the subsequent experiment. Proteins were extracted from skin tissues using a lysis buffer. The total protein concentration was then determined using a BCA protein assay kit. Equal amounts of protein were separated on sodium dodecyl sulfate‐polyacrylamide gel electrophoresis (7.5%–12.5% SDS‐PAGE), and then transferred to a polyvinylidene fluoride (PVDF) membrane (Bio‐Rad, 1620177). The membrane was blocked with 5% skim milk in TBST at room temperature for 1.5 h, and subsequently incubated with the following primary antibodies against overnight at 4°C: ACSL4 (1:5000, 22401‐1‐AP, Proteintech), XCT (1:1000, 26864‐1‐AP, Proteintech), GPX4 (1:1000, ab125066, Abcam), GAPDH (1:5000, 301341, Zen bio), NOX2 (1:1000, DF6520, Affinity), COX2 (1:1000, AF7003, Affinity), LATS1 (1:1000, sc‐398560, Santa), LATS2 (1:1000, 20276‐1‐AP, Proteintech), YAP (1:1000, sc‐101199, Santa), Histone‐H3 (1:1000, 17168‐1‐AP, Proteintech), TNF‐α (1:1000, 17590‐1‐AP, Proteintech), HO‐1 (1:1000, 10701‐1‐AP, Proteintech), CD206 (1:1000, 18704‐1‐AP, Proteintech) and CD86 (1:1000, 13395‐1‐AP, Proteintech). After three washes with TBST, the membranes were incubated with HRP‐conjugated IgG secondary antibodies for 1 h at ambient temperature. Finally, the bands were visualised using ChemiDic XRS+ Imaging System (Bio‐Rad, USA), and quantified by Image J.

### 2.5 Perl's Iron Staining

The 5‐μm‐thick skin sections from full‐thickness wounds were deparaffinised in xylene I and II (15 min each) and hydrated through graded alcohol solutions (100%, 95%, 85%, and 75%, 5 min each). Sections were stained using a Perl's iron staining kit (Solarbio, G1424) according to the manufacturer's protocol, briefly dehydrated in ethanol, cleared in xylene and mounted with resin. Perl's‐stained areas (blue) were imaged under a bright‐field microscope (Nikon, Japan) and quantified using Image J's thresholding method.

### 2.6 MDA Measurement

The relative MDA concentration in skin tissues was evaluated with a lipid peroxidation MDA assay kit (S0131S, Beyotime), on the basis of the manufacturer's instructions. Briefly, skin tissues were homogenised in lysis buffer, incubated with thiobarbituric acid at 100°C for 15 min, centrifuged, and then absorbance was measured at 532 nm.

### 2.7 Iron Content

Samples were collected 7 days after modelling. The iron content was measured using a iron assay kit (bc4355, Solarbio) according to the manufacturer's instructions. The iron content in the samples was evaluated by a spectrophotometer at 520 nm. Then the iron content in the tissues was calculated using the corresponding formula.

### 2.8 Reduced Glutathione (GSH) Measurement

To measure GSH and GSSG levels, samples collected 7 days post‐modelling were homogenised at 4°C. Using a GSH/GSSG Assay Kit (Beyotime, S0053), GSSG was reduced to GSH by glutathione reductase. The GSH then reacts with 2‐nitrobenzoic acid (DTNB), producing a yellow‐coloured 5‐thio‐2‐nitrobenzoic acid (TNB). TNB was quantified spectrophotometrically at 420 nm. The results were normalised to protein concentration.

### 2.9 Statistical Analysis

The data in the graph were expressed as means ± SD. The results from the experimental groups were compared using Student's *t*‐test to calculate *p*‐values with GraphPad prism 9.0 software. Values of *p* < 0.05 was considered statistically significant.

## 3 Results

### 3.1 CuATSM Improves Wound Healing in Mice

**FIGURE 1:**
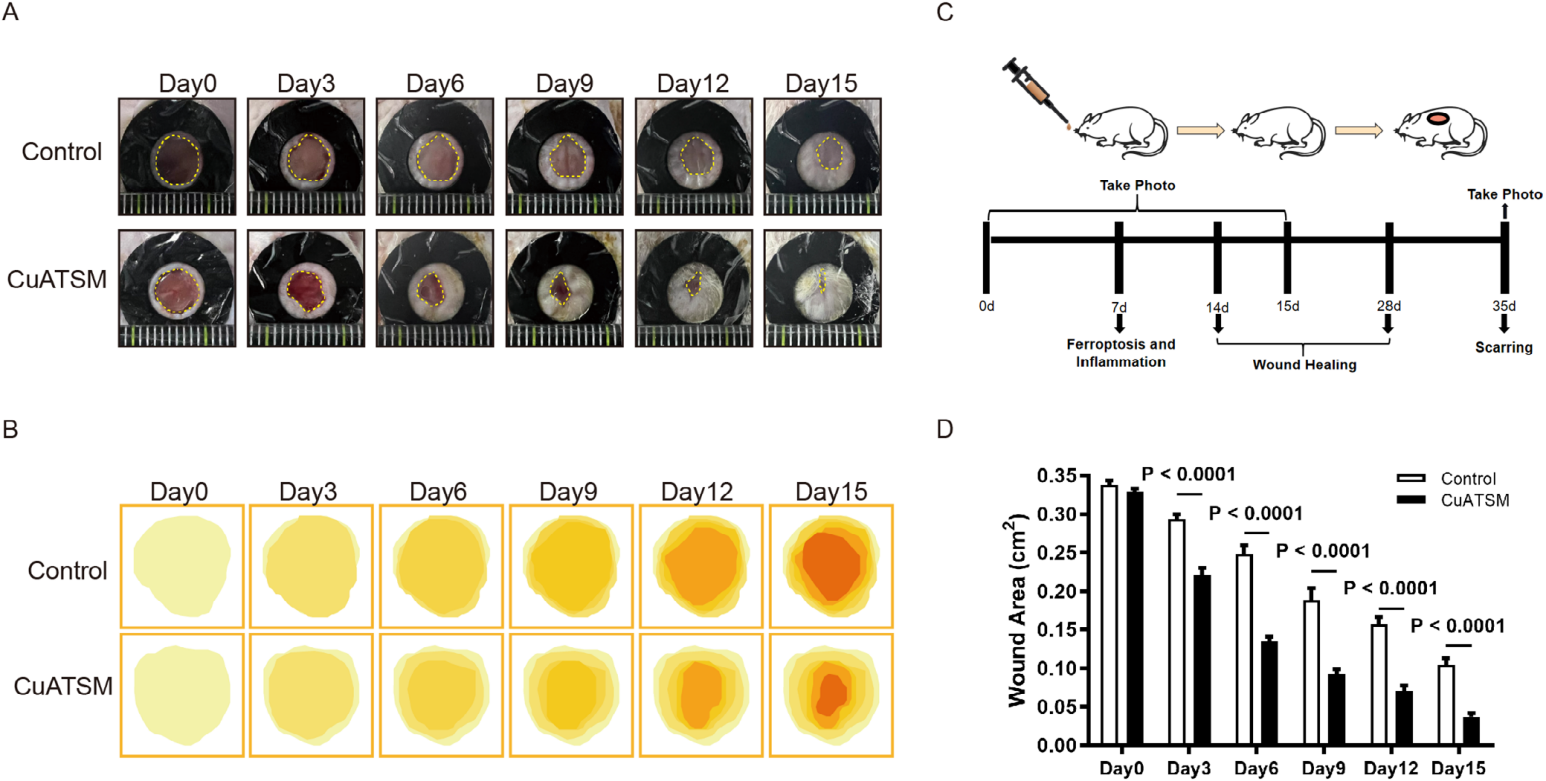
CuATSM improves wound healing in mice. (A) The healing condition of skin wounds in Control and CuATSM groups of mice at days 0, 3, 6, 9, 12 and 15; (B) Simulating the range of wounds based on wound images to visually display the contours of the wounds; (C) Experimental flowchart; (D) Statistical analysis of wound area at different time points for Control and CuATSM groups, *p* < 0.0001, *n* = 10.

To assess the effects of CuATSM on skin wound healing in mice, we performed a full‐thickness skin excision model. Specifically, circular wounds approximately 6 mm in diameter were created on the dorsal region of the mice, which were then randomly assigned to two distinct groups: a CuATSM‐treated group and a Control group. Wound images were captured on days 0, 3, 6, 9, 12 and 15 post‐injury to monitor the healing process. Following CuATSM treatment, a significant reduction in wound area was observed from day 3 onward. By day 15, the wounds in the CuATSM group showed substantial healing compared to the Control group, as illustrated in Figure 1A,B. Consistent with these findings, statistical analysis revealed a significant reduction in wound area in the CuATSM group compared to the Control group from day 3 onward, with faster healing progression maintained throughout (Figure 1D). Together, these findings indicate that CuATSM markedly enhances the wound healing process.

### 3.2 CuATSM Accelerates Granulation Tissue Formation and Enhances Collagen Deposition in Mouse Skin Wounds

**FIGURE 2:**
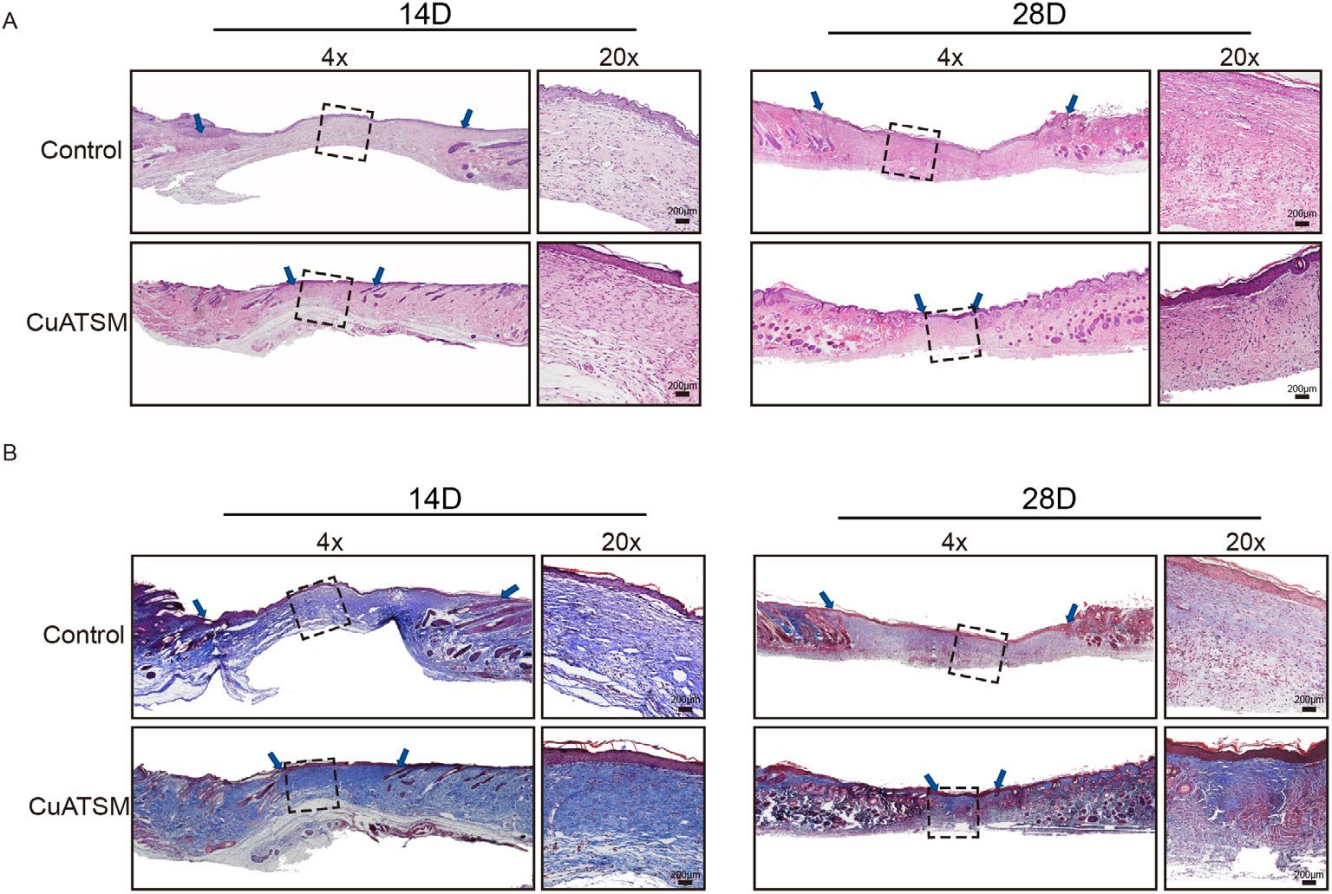
CuATSM accelerates granulation tissue formation and enhances collagen deposition in mouse skin wounds. (A, B) Haematoxylin and Eosin (H&E) staining and Masson's trichrome staining in days 14 and 28. scale bar = 200 μm, *n* = 4.

To gain deeper insights into the microstructural dynamics and the overall progression of healing, we conducted meticulous histological staining analyses on mouse skin wounds at crucial time points of day 14 and 28. Granulation tissue formation during the healing process was assessed across different groups using H&E staining. Our findings revealed that the CuATSM‐treated wounds exhibited a significantly greater abundance of granulation tissue when compared with the Control group. Furthermore, the CuATSM group consistently demonstrated narrower widths of granulation tissue, highlighting the potent stimulatory effect of CuATSM on granulation tissue generation (Figure 2A). Moreover, collagen deposition, a crucial aspect of early skin wound repair, was evaluated using Masson's trichrome staining. The CuATSM‐treated wounds showed a significantly more intense blue staining at both days 14 and 28, indicating enhanced collagen deposition (Figure 2B). In conclusion, CuATSM not only accelerates the regeneration of granulation tissue during skin wound repair but also augments collagen deposition, thereby enhancing the overall wound healing process.

### 3.3 CuATSM Enhances Angiogenesis, Cellular Proliferation and Collagen Deposition During Cutaneous Wound Healing in Mice

**FIGURE 3:**
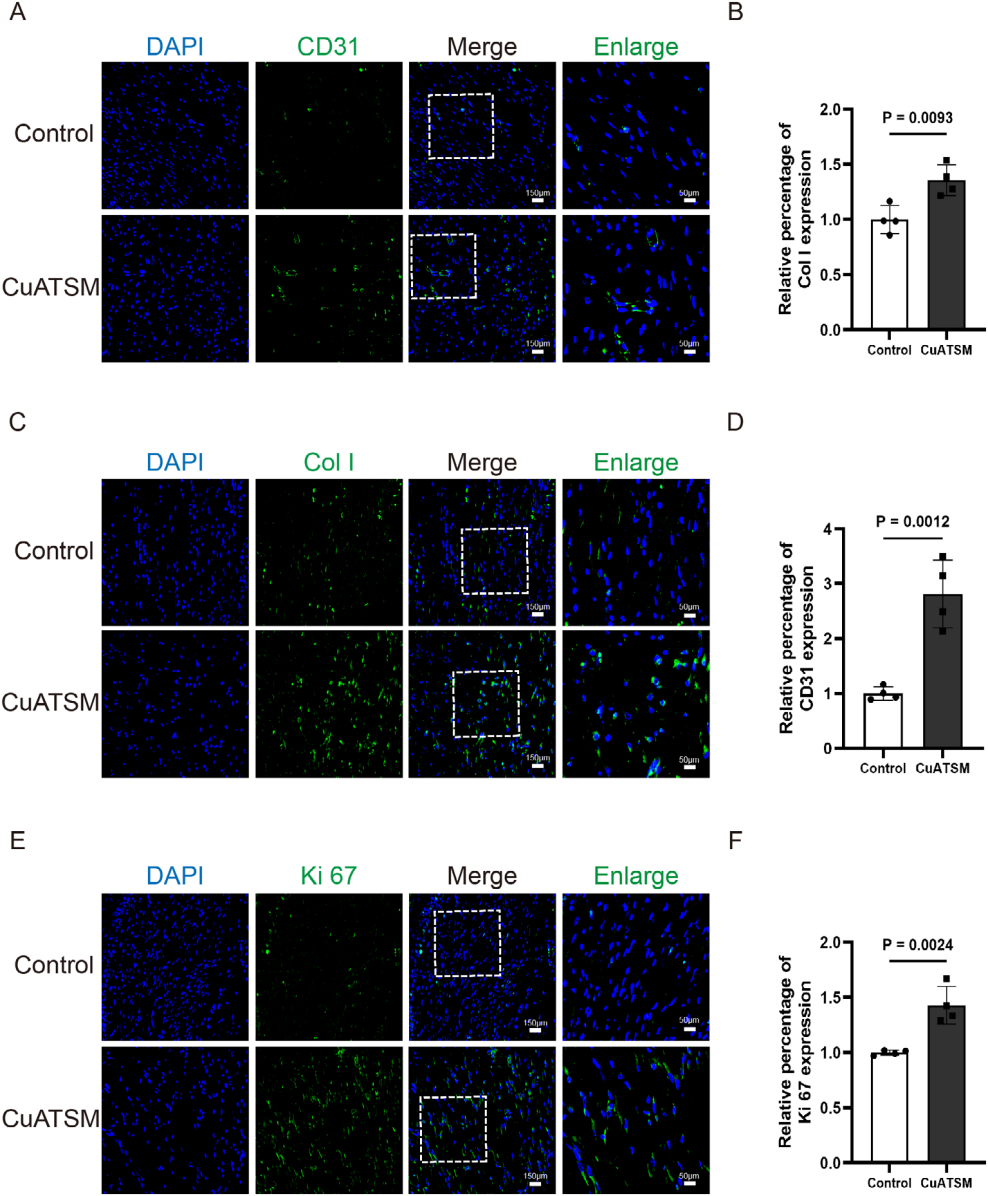
CuATSM enhances angiogenesis, cellular proliferation and collagen deposition during cutaneous wound healing in mice. (A) Representative fluorescence images of skin stained with CD31 (green) and DAPI (blue) in mice from the two groups at day 7 after full‐thickness skin excision, digitalized pictures were selected in the central regions, scale bars = 150 μm, enlarge scale bars = 50 μm; (B) Relative fluorescence intensity of CD31, CD31 presence significantly differed between the two groups, *p* = 0.0093, *n* = 4; (C) Representative fluorescence images of skin stained with Col I (green) and DAPI (blue) in mice from the two groups at day 7 after full‐thickness skin excision, digitalized pictures were selected in the central regions, scale bars = 150 μm, enlarge scale bars = 50 μm; (D) Relative fluorescence intensity of Col I, Col I presence significantly differed between the two groups, *p* = 0.0012, *n* = 4; (E) Representative fluorescence images of skin stained with Ki67 (green) and DAPI (blue) in mice from the two groups at day 7 after full‐thickness skin excision, digitalized pictures were selected in the central regions, scale bars = 150 μm, enlarge scale bars = 50 μm; (F) Relative fluorescence intensity of Ki67, Ki67 presence significantly differed between the two groups, *p* = 0.0024, *n* = 4.

To evaluate the angiogenic potential in wound tissues, we utilised CD31, a specific marker for endothelial cells, to analyse vascular expression patterns and distribution. Immunofluorescence staining for CD31 (Figure 3A,B) showed robust neovascularisation in CuATSM‐treated wounds on day 7 post‐wounding, with a marked increase in vascular density compared to the control group, revealing its stimulatory effect on angiogenesis during cutaneous wound healing. Meanwhile, immunofluorescence staining was performed on day 7 to assess the expression of Col I and Ki67 in wound tissues. As shown in Figure 3C,D, CuATSM treatment significantly increased Col I deposition compared to the Control group. Additionally, CuATSM markedly upregulated Ki67 expression, as depicted in Figure 3E,F, indicating enhanced cellular proliferation within the wound bed. Collectively, these findings highlight the therapeutic potential of CuATSM in enhancing cellular proliferation, promoting Col I synthesis and angiogenic processes, thereby facilitating cutaneous wound healing in a murine model.

### 3.4 CuATSM Enhances Scarless Wound Healing In Vivo

**FIGURE 4:**
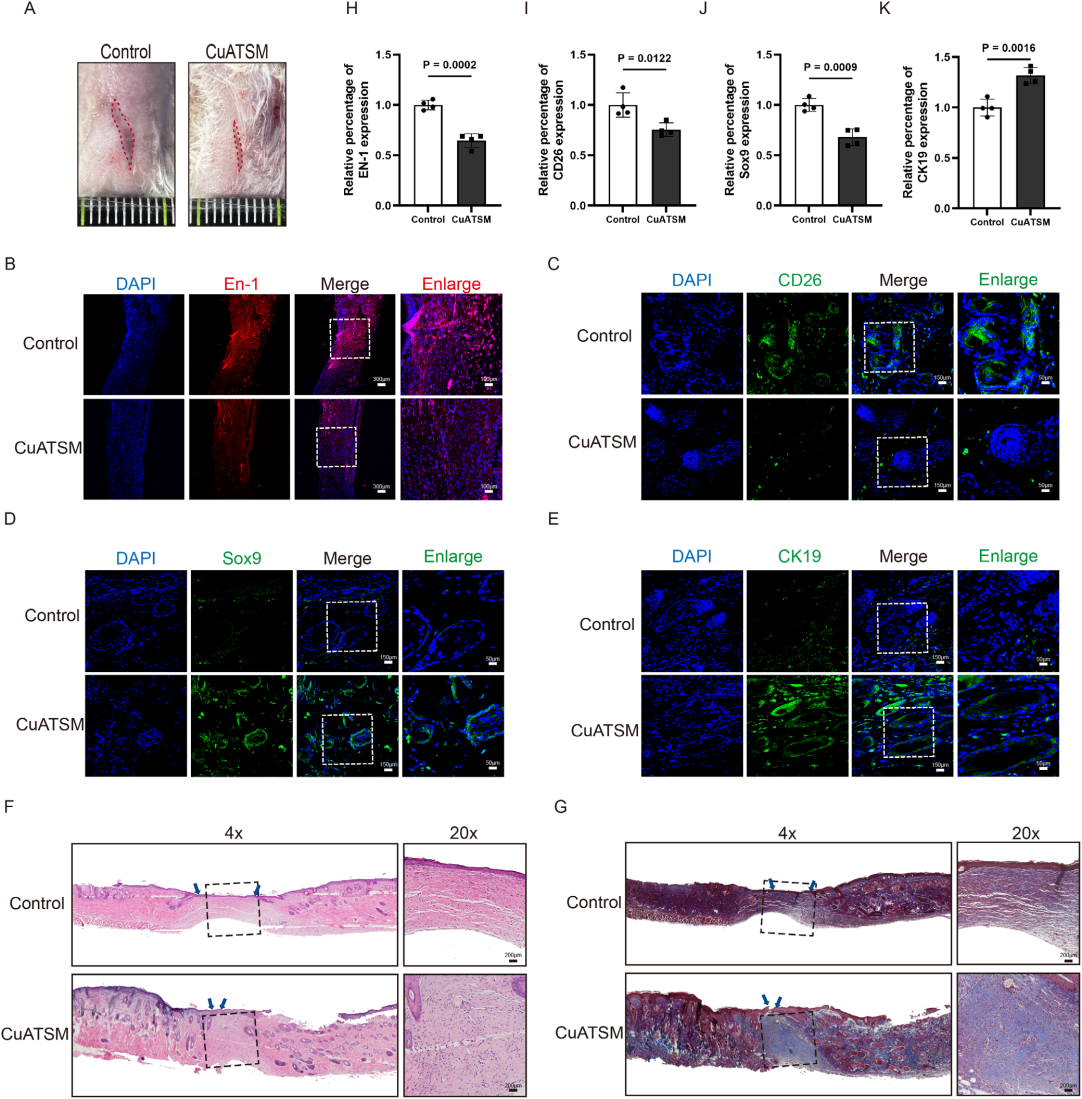
CuATSM enhance scarless wound healing in vivo. (A) On day 35, the healing and hair growth of skin wounds in mouse from the Control group and the CuATSM group, *n* = 3; (B–E) Representative fluorescence images of skin stained with En‐1 (red) and DAPI (blue) in mice from the two groups at day 35 after full‐thickness skin excision, scale bars = 300 μm, enlarge scale bars = 100 μm; Representative fluorescence images of skin stained with CD26 (green), Sox9 (green) and CK19 (green) and DAPI (blue) in mice from the two groups at day 35 after full‐thickness skin excision, digitalized pictures were selected in the central regions, scale bars = 150 μm, enlarge scale bars = 50 μm; (H–K) Relative fluorescence intensity of En‐1, CD26, Sox9 and CK19, En‐1, CD26, Sox9 and CK19 presence significantly differed between the two groups *p* = 0.0002, 0.0122, 0.0009 and 0.0016, respectively; *n* = 4; (F, G) Haematoxylin and Eosin (H&E) staining and Masson's trichrome staining in the 35th day, scale bar = 200 μm, *n* = 4.

To evaluate the effect of CuATSM on scar formation during skin wound healing, we examined the wounds on the 35th day post‐injury and performed histological staining on the skin tissue. Compared to the Control group, the CuATSM‐treated group exhibited significantly improved healing, accompanied by denser hair regrowth (Figure 4A). H&E staining and Masson staining further confirmed a substantial enhancement in hair follicle formation and structural integrity in the CuATSM‐treated group on day 35 (Figure 4F,G). Previous studies have demonstrated that inhibiting the activation of Engrailed‐1 (En1) and dipeptidyl peptidase 4 (DPPIV/CD26) in fibroblasts can promote scarless wound healing [28, 29]. The expression of CD26‐positive and En‐1‐positive cells in the CuATSM‐treated skin tissue was significantly lower than that in the Control group (Figure 4B,C,H,I). Hair follicle regeneration is a critical component of effective wound healing, and hair follicle stem cells play a pivotal role in this process. These stem cells are characterised by the expression of cytokeratin 19 (CK19) and the transcription factor sex‐determining region Y‐box 9 (Sox9) [30, 31]. Thus, we performed immunofluorescence staining for CK19 and Sox9 on the skin tissue (Figure 4D,E,J,K). These results showed that the CuATSM‐treated group had a significantly higher abundance of CK19‐positive and Sox9‐positive cells than the Control group. Based on the above results, CuATSM promotes scarless wound healing by inhibiting scar formation and enhancing hair follicle regeneration.

### 3.5 CuATSM Inhibits Ferroptosis During Wound Healing

**FIGURE 5:**
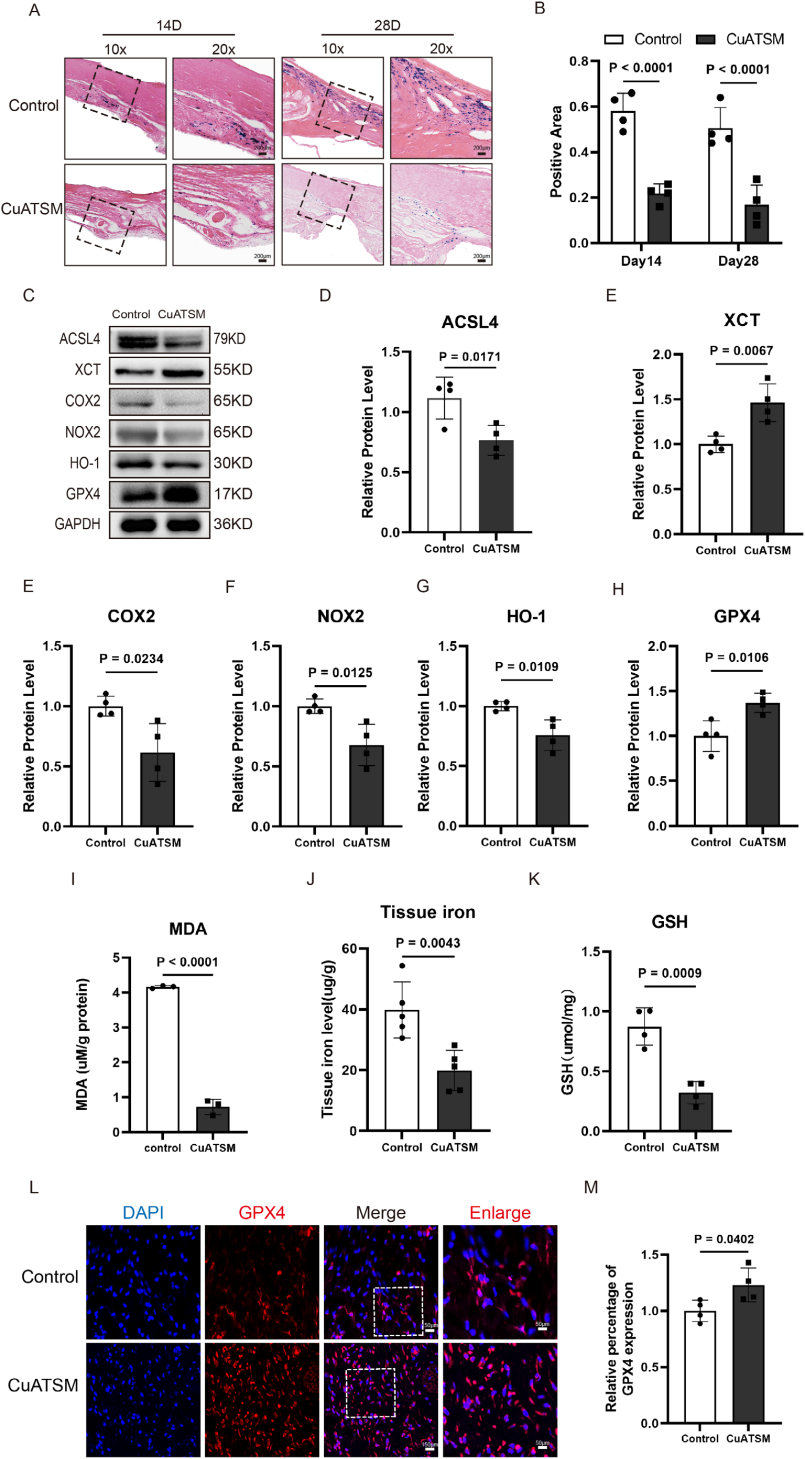
CuATSM inhibits ferroptosis during wound healing. (A) Perl's iron staining in days 14 and 28, scale bar = 200 μm; (B) Iron‐stained positive area presence significantly differed between the two groups, *p* < 0.0001, *n* = 4; (C) Western blotting was used to detect the expression of ferroptosis‐related proteins; (D–H) Quantitative analysis of the expression levels of related proteins in the CuATSM group compared to the Control group, *p* = 0.0171, 0.0067, 0.0234, 0.0125, 0.0109 and 0.0106; *n* = 4. (I) The MDA content in skin wound tissues of mice in the Control and CuATSM groups on day 7, *p* < 0.0001, *n* = 3; (J) The tissue iron content in skin wound tissues of mice in the Control and CuATSM groups on day 7, *p* = 0.0043, *n* = 5; (K) The GSH content in skin wound tissues of mice in the Control and CuATSM groups on day 7, *p* = 0.0009, *n* = 4; (L) Representative fluorescence images of skin stained with GPX4 (red) and DAPI (blue) in mice from the two groups at day 7 after full‐thickness skin excision, digitalized pictures were selected in the central regions, scale bars = 150 μm, enlarge scale bars = 50 μm; (M) Relative fluorescence intensity of GPX4, GPX4 presence significantly differed between the two groups, *p* = 0.0402, *n* = 4.

To further investigate the effect of CuATSM on ferroptosis in skin wounds, we utilised Perl's iron staining to examine mouse skin tissue on days 14 and 28 post‐injury. Compared to the Control group, the CuATSM group exhibited a significant reduction in iron‐positive cells (Figure 5A,B). To confirm whether CuATSM can inhibit iron‐mediated cell death in skin wounds, we performed Western blot for ferroptosis‐related proteins in mouse skin wound tissue on the 7th day. As shown in Figure 5C–H, CuATSM decreased the expression of the proteins ACSL4, HO‐1, COX2 and NOX2 while simultaneously enhancing the expression of XCT and GPX4 in the tissue on the 7th day after full‐thickness skin excision surgery in mice. Next, we performed immunofluorescence staining for GPX4 to further explore the distribution of it in the skin tissue. The results indicated that the number of GPX4‐positive cells in the CuATSM group was increased when compared to the Control group (Figure 5L,M). Additionally, we measured the levels of glutathione (GSH) and malondialdehyde (MDA) in the skin tissue. Malondialdehyde (MDA), a highly reactive small molecule produced by lipid peroxidation, is a marker of oxidative stress [32]. We found that CuATSM inhibited the upregulation of MDA levels in the skin tissue on the 7th day (Figure 5I). GSH plays a crucial role in protecting against ferroptosis [33]. Notably, we found that the GSH levels were significantly elevated in the CuATSM group compared to the Control group (Figure 5K). Furthermore, we measured the tissue iron content in the wound tissue of each group on the 7th day and discovered a significant reduction in the CuATSM group (Figure 5J). These data demonstrate that ferroptosis can be activated after skin injury, and CuATSM treatment effectively inhibits ferroptosis and thereby promotes wound healing.

### 3.6 CuATSM Promotes Macrophage Polarisation, Attenuating Inflammatory Responses and Enhancing Cutaneous Wound Healing

**FIGURE 6:**
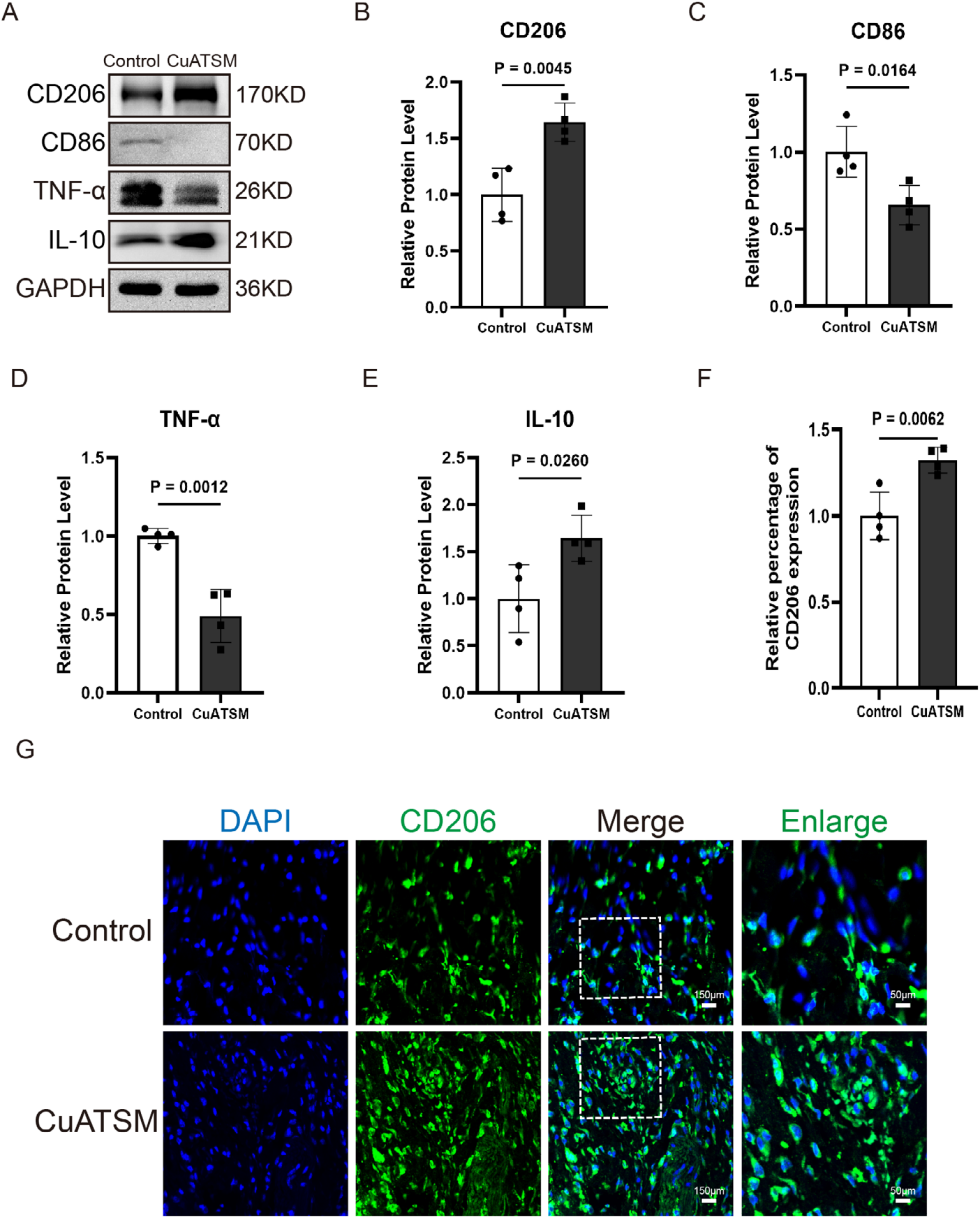
CuATSM promotes macrophage polarisation, attenuating inflammatory responses and enhancing cutaneous wound healing. (A) Western blotting was used to detect the expression of macrophage‐related proteins and inflammatory factors; (B–E) Quantitative analysis of the expression levels of related proteins in the CuATSM group compared with the Control group. *p* = 0.0045, 0.0164, 0.0012 and 0.0260, *n* = 4 ; (F) Relative fluorescence intensity of CD206, CD206 presence significantly differed between the two groups, *p* = 0.0062, *n* = 4; (G) Representative fluorescence images of skin stained with CD206 (green) and DAPI (blue) in mice from the two groups at day 7 after full‐thickness skin excision, digitalized pictures were selected in the central regions, scale bars = 150 μm, enlarge scale bars = 50 μm.

We further evaluated whether CuATSM could promote wound regeneration by regulating macrophage polarisation. In this study, the expression of M1 and M2 markers in mice was investigated by Western blot and immunofluorescence chemical staining on day 7 post‐treatment to reveal the polarisation status of macrophages during wound healing. In the CuATSM treatment group, the expression levels of CD86 (a marker for M1 phenotype) were decreased, while the expression levels of CD206 (a marker for M2 phenotype) were increased compared with the control group (Figure 6A–C). Moreover, the expression of pro‐inflammatory cytokines such as TNF‐α was reduced, while the expression of anti‐inflammatory cytokine such as IL‐10 was increased in the CuATSM‐treated group (Figure 6D,E). In addition, immunofluorescence analysis showed that CuATSM treatment induced a substantial increase in CD206 expression in wound tissues (Figure 6G,F). These results suggest that CuATSM promotes macrophage polarisation toward the M2 phenotype, thereby regulating inflammation and accelerating early wound healing.

### 3.7 CuATSM Modulates Hippo/YAP Pathway During Wound Healing

**FIGURE 7:**
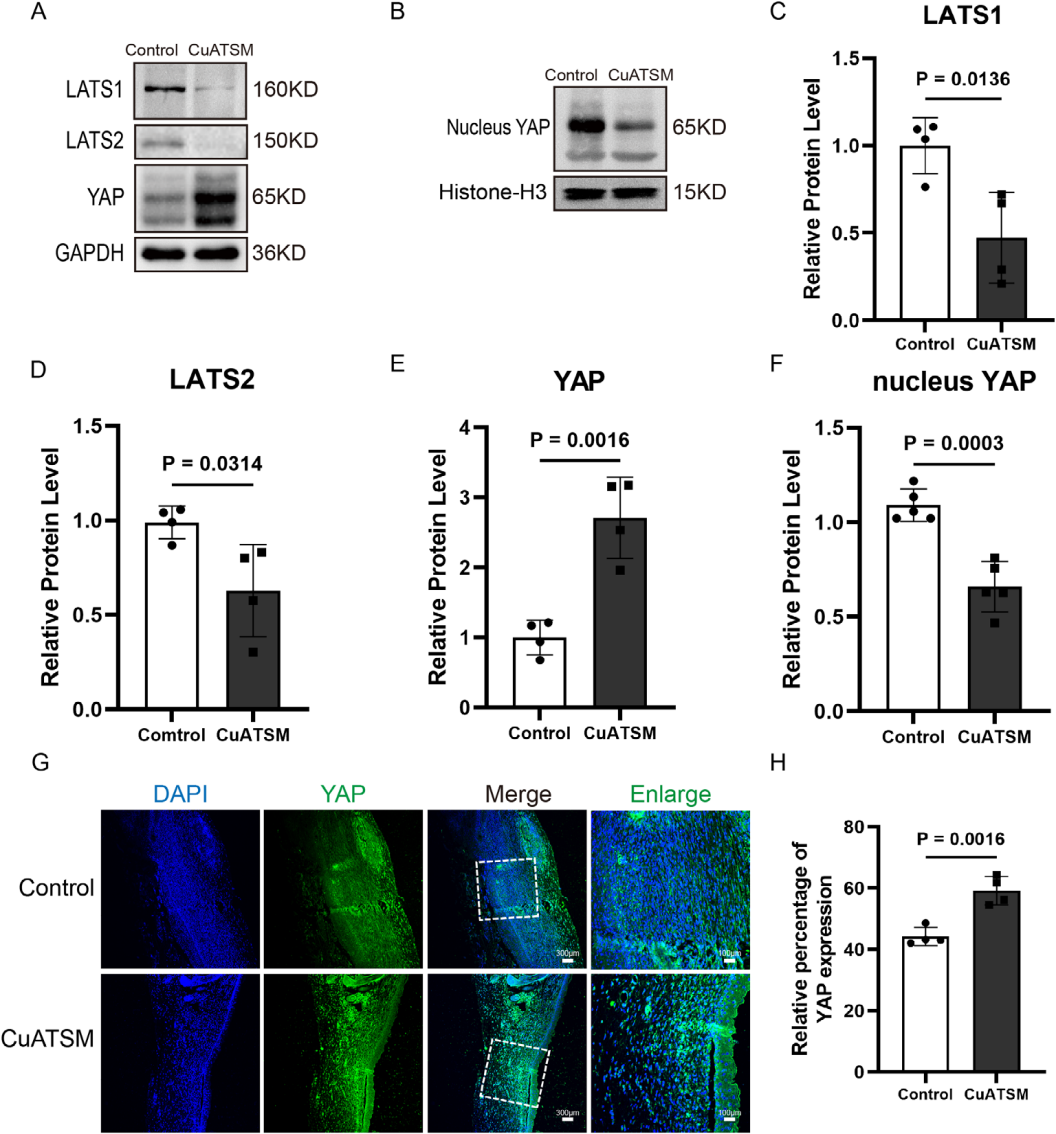
CuATSM modulates Hippo/YAP pathway during wound healing. (A) Western blotting was used to detect the expression of Hippo‐related proteins outside the nucleus; (B) Western blotting was used to detect the expression of YAP within the nucleus; (C–F) Quantitative analysis of the expression levels of related proteins in the CuATSM group compared to the Control group, *p* = 0.0136, 0.0314, 0.0016, and 0.0003; *n* = 4; (G) Representative fluorescence images of skin stained with YAP (green) and DAPI (blue) in mice from the two groups at day 35 after full‐thickness skin excision, scale bars = 300 μm, enlarge scale bars = 100 μm; (H) Relative fluorescence intensity of YAP, YAP presence significantly differed between the two groups, *p* = 0.0016, *n* = 4.

The Hippo/YAP signalling pathway plays a crucial role in the proliferation of scars [14]. To investigate the role of CuATSM in the Hippo/YAP signalling pathway, we performed western blotting in skin tissue on the day 7 to evaluate the expression levels of Hippo/YAP‐related proteins in wound tissue. Compared to the Control group, the protein expression levels of LATS1 and LATS2 were significantly decreased after CuATSM treatment, while the expression of YAP protein in the cytoplasm was significantly increased (Figure 7A,C,D,E). Additionally, we examined the protein expression levels of YAP in the cell nucleus, and the results revealed that CuATSM significantly inhibited the protein expression of YAP in the cell nucleus (Figure 7B,F). Meanwhile, we used immunohistochemical staining to detect the distribtion of YAP in the skin tissue. The number of YAP‐positive cells in the CuATSM group was significantly higher than that in the Control group (Figure 7G,H). These results indicate that CuATSM regulates wound healing by adjusting the Hippo/YAP signalling pathway.

## 4 Discussion

Despite significant advances in science and evidence‐based medicine, scar formation during wound repair remains a major concern. Thus, there is an urgent need to find a drug that effectively inhibits scar development in the early stages of wound healing. In the present study, we treated mice with CuATSM and found that CuATSM not only inhibits scarring but also promotes wound repair. Mechanistic investigations further revealed that the protective effects of CuATSM on wound repair and its promotion of scarless wound healing are associated with modulation of the Hippo/YAP signalling pathway, macrophage polarisation and ferroptosis suppression. Furthermore, CuATSM further promotes wound repair through Hippo pathway‐dependent ferroptosis suppression, synergistically enhancing tissue regeneration and mitigating pathological fibrosis.

CuATSM, a positron emission tomography (PET) tracer with hypoxia sensitivity, has been proven effective in several neurodegenerative diseases, including amyotrophic lateral sclerosis (ALS), Parkinson's disease, ischemic stroke and neuroinflammation [34, 35, 36]. In this study, we observed a significant acceleration in the wound healing rate from the third day onward in mice treated with CuATSM, with a notably larger healing area compared to the Control group by capturing wound photographs. Histological analyses performed on days 14 and 28 revealed that CuATSM treatment significantly enhanced granulation formation in the wounds. Additionally, masson staining results indicated that skin wound tissues treated with CuATSM exhibited greater collagen deposition on days 14 and 28. Furthermore, immunofluorescence analysis of CoI I showed significantly higher expression of Col I in the CuATSM‐treated group compared to the Control group. To further evaluate cellular proliferation, we investigated Ki67, a nuclear protein associated with cell division activity. Immunofluorescence staining of skin wound tissue showed a significant increase in Ki67‐positive cells in the CuATSM‐treated group compared to the control group. Meanwhile, the formation of new blood vessels is critical for wound healing as they provide essential oxygen and nutrients required for tissue metabolism and cellular processes [37]. Using immunofluorescence staining for the blood vessel marker CD31, we observed a more extensive and intricate vascular network in the wound tissue of the CuATSM group compared to the control group. In summary, our findings suggest that CuATSM significantly promotes wound healing by increasing collagen deposition, enhancing cellular proliferation and improving angiogenesis, positioning it as a potential drug for tissue repair and regeneration.

Recently, CuATSM has emerged as a promising inhibitor of ferroptosis [23]. Ferroptosis, a recently recognised form of programmed cell death (PCD), is characterised by metabolic dysregulation of intracellular lipid peroxides, which are catalysed by iron ions to generate excessive reactive oxygen species (ROS), ultimately leading to cell death [38]. This process is closely linked to inflammation and is involved in the development and progression of various diseases [39]. CuATSM can inhibit ferroptosis and reduce lipid peroxidation, thereby protecting primary and immortalised neuronal cells [40]. Previous studies demonstrated that ferroptosis activation is involved in the progression of diabetic wound repair, indicating that Fer‐1, a ferroptosis inhibitor, could accelerate healing of diabetic skin wounds in rats by upregulating the PI3K/Akt signalling pathway [41]. Based on these studies, our study is the first to demonstrate that the protective effect of CuATSM on wound repair is directly associated with the downregulation of ferroptosis. Our results indicate that CuATSM effectively reversed iron accumulation in the injured skin tissue. Meanwhile, CuATSM down‐regulated the expression of ACSL4, HO‐1, COX2 and NOX2 while up‐regulated the expression of XCT and GPX4 when compared to the Control group, which was consistent with a previous study. Furthermore, we detected the changes in glutathione and lipid peroxidation derivative MDA content and found that, in contrast to the Control group, the GSH content in the skin wound tissue treated with CuATSM was notably increased, and the MDA content was significantly diminished. This may be attributed to CuATSM's capacity to capture free radicals, exhibit antioxidant properties and inhibit lipid peroxidation.

Ideal wound healing involves not only rapid early recovery but also minimal scar formation in the later stages [42]. A crucial process of early healing is macrophage polarisation. Macrophages can differentiate into M1‐type and M2‐type macrophages. M1 macrophages exacerbate inflammation through the release of pro‐inflammatory cytokines, leading to tissue dysfunction, while M2 macrophages secrete anti‐inflammatory factors that help mitigate inflammation and promote wound healing. However, poorly healing wounds are often accompanied by dysregulated macrophage polarisation, leading to an inflammatory storm [43]. Previous studies suggest that CuATSM treatment significantly reduced the protein expression of pro‐inflammatory cytokines in the neointimal tissue [44]. Our study demonstrated that CuATSM treatment significantly upregulated anti‐inflammatory markers such as IL‐10 and CD206, while downregulating pro‐inflammatory markers like TNF‐α and CD86, compared to the Control group. Immunofluorescence staining further revealed that CuATSM increased the number of CD206‐positive cells on day 7 post‐injury. These findings suggest that CuATSM accelerates wound healing by promoting M2 macrophage polarisation and reducing inflammation. Besides inflammation control, the prevention of scar formation is critical for ideal wound healing. Recent studies have highlighted the importance of hair follicles and their stem cells in scarless wound healing [45]. Several studies have shown that hair follicles possess characteristics such as promoting tissue regeneration, regulating immune responses, reducing fibrosis and enhancing angiogenesis, making them one of the key targets in research and clinical treatments for scarless wound healing [46]. In this study, the CuATSM group showed a significant increase in the number and integrity of hair follicles on day 35. Immunofluorescence revealed that CuATSM enhanced the expression of hair follicle stem cell markers (CK19, Sox9), indicating that CuATSM can promote hair follicle regeneration and the formation of hair follicle stem cells during the later stages of wound healing, thereby reducing scarring during the healing process. Furthermore, fibroblasts, particularly those expressing En1, named En1 lineage‐positive fibroblasts (EPF) are involved in extracellular matrix (ECM) secretion and scar tissue formation [47]. By inhibiting these fibroblasts, scarless healing can be facilitated [48]. Fibroblasts expressing CD26 have been identified as a primary cellular source of ECM in the skin [49, 50]. Rinkevich et al. found that inhibiting CD26 significantly reduces scar formation during the wound healing process [51]. Furthermore, existing studies suggest that either genetic knockout of the DPP4 gene or pharmacological inhibition of DPP4 can promote scarless healing in murine skin [51]. In our study, immunofluorescence staining of mouse skin wounds at day 35 post‐injury revealed a significant reduction in the number of En‐1 and CD26‐positive cells in the wound tissue of the CuATSM‐treated group compared to the Control group. These results indicate that CuATSM effectively reduces the population of fibroblasts involved in scar formation, thereby facilitating scarless wound healing.

The Hippo signalling pathway, an evolutionarily conserved cascade, is critically involved in regulating immune responses, wound healing and tissue regeneration. Numerous studies from diverse organ systems demonstrate the important roles that YAP and TAZ play in promoting fibroblast activation [52, 53, 54]. In 2021, a study published in Science demonstrated that the knockout of the YAP gene inhibits the activation of En1 in fibroblasts, leading to enhanced proliferation of En1‐negative fibroblasts (ENF), which promotes wound healing. Meanwhile, the reduction in the generation of EPF results in decreased scar formation [48]. Mengfan Wu et al. found that the application of negative pressure therapy on mouse skin wounds increased the number of YAP‐positive cells while significantly reducing the fibroblast scar proliferation marker CD26 [55]. These findings suggest that the regulation of the Hippo/YAP pathway in fibroblasts is closely associated with scarless wound healing. Our study demonstrated that CuATSM treatment effectively downregulates the protein expression of upstream YAP regulators, LATS1 and LATS2, which are key components of the Hippo signalling pathway. We observed that the CuATSM reduced nucleus YAP protein levels. In contrast, CuATSM treatment led to a concurrent increase in cytoplasmic YAP protein levels. Meanwhile, immunofluorescence staining revealed a significant increase in the number of YAP‐positive cells in the CuATSM‐treated group compared to the Control group. These results indicate that CuATSM may modulate the Hippo/YAP signalling pathway by inhibiting YAP nuclear translocation, thereby promoting scarless wound healing.

It has been reported that YAP promotes ferroptosis by upregulating multiple ferroptosis modulators, including ACSL4 and transferrin receptor (TFRC), which mediate lipid peroxidation and iron overload, respectively [56, 57]. In this study, we found that CuATSM increases cytoplasmic YAP protein levels while reducing its nuclear expression. CuATSM also down‐regulates the expression of ACSL4. Building on the established role of YAP/TAZ in regulating ferroptosis, we propose that CuATSM suppresses ferroptosis and accelerates cutaneous wound healing through inhibition of the YAP‐ACSL4 axis. Specifically, CuATSM blocks YAP nuclear localization and thereby reduces ACSL4 transcription, consequently suppressing ferroptosis and enhancing tissue repair in skin. Unfortunately, the Hippo/YAP signalling pathway also encompasses important upstream regulators such as MST1/2 and downstream effectors like the YAP/TAZ/TEAD complex [58, 59], which were not further investigated in the present study. We acknowledge this limitation and hope to explore these components in future research to provide a more comprehensive understanding of the pathway. Given the established role of YAP as the rate‐limiting node in this pathway, we prioritised mechanistic interrogation of this target. However, potential crosstalk with upstream effectors remains an important avenue for subsequent validation.

In summary, CuATSM enhances wound healing by promoting granulation tissue formation, collagen deposition, angiogenesis and cell proliferation. In early repair stages, CuATSM drives macrophage polarisation toward the M2 phenotype, reduces inflammation and decreases ferroptosis by lowering lipid peroxides and exerting antioxidant effects. During later stages, CuATSM stimulates hair follicle regeneration and stem cell activation while inhibiting excessive fibroblast proliferation and scar formation. Furthermore, CuATSM regulates the Hippo/YAP pathway, preventing YAP nuclear translocation and minimising scar formation. This study highlights CuATSM as a promising candidate for clinical applications targeting scarless skin regeneration.

## Author Contributions

**Yingdan Tang:** data curation (lead), formal analysis (lead), investigation (equal), methodology (lead), software (equal), validation (equal), visualization (equal), writing – original draft (equal). **Jiazong Ye:** data curation (equal), investigation (equal), methodology (equal). **Tingye Xu:** data curation (lead), formal analysis (equal), methodology (lead), software (equal). **Yuhuan Sun:** data curation (lead), formal analysis (equal), methodology (equal). **Weiyang Meng:** data curation (equal), formal analysis (equal), investigation (equal), methodology (equal). **Lielie Zhu:** data curation (equal), formal analysis (equal), investigation (equal). **Zhongheng Jia:** data curation (equal), formal analysis (equal), investigation (equal), methodology (equal). **Qian Wu:** conceptualization (equal), validation (equal), visualization (equal). **Daqing Chen:** conceptualization (equal), funding acquisition (equal), project administration (equal), supervision (equal). **Fangfang Wu:** conceptualization (lead), funding acquisition (equal), project administration (lead), supervision (lead), writing – original draft (lead), writing – review and editing (lead).

## Conflicts of Interest

The authors declare no conflicts of interest.

## Data Availability

The data that support the findings of this study are available from the corresponding author upon reasonable request.
